# Lectures on Dentistry for Popular Audiences

**Published:** 1887-07

**Authors:** 


ARTICLE III.

### LECTURES ON DENTISTRY FOR POPULAR AUDIENCES.

All dentists of long experience have noted again and again how bitterly people regret the heedlessness of themselves or their parents, which has resulted for themselves in a ruined set of teeth, an enfeebled digestion, and all the chain of attendant evils which such a state of things necessarily brings about. "I cannot," such a one will say, "altogether blame myself for my neglect of my teeth; when I was a child I knew no better; indeed, I never realized how far the well-being of my poor teeth depended upon my care." And small wonder. Who, outside the profession, can appreciate the slight initial indications of decay? To most persons, save those who have been brought frequently into intercourse with their dentists, ignorance supreme about the physiology of dentition is the rule; they also know nothing of the measures necessary to induce healthy growth of the teeth; and further, are equally unable to direct reasonable endeavors towards maintaining their organs of mastication free from disease. As a rule, persons wait for the advent of toothache to tell them something is wrong with their teeth, and when this occurs, if children, their idea is to get the tooth pulled out—a plan too often acquiesced in by their badly-informed parents. Any measure, then, which gives hope that some instruction will be given in untechnical terms to untechnical minds in the many matters concerned with the growth, health and caretaking of the teeth, should be welcomed with pleasure alike by the dental profession and the public. We mentioned very briefly in our last issue that Mr. Henry Moxon, as dental surgeon in charge of the Anerley Schools, had undertaken a departure in this direction. Handling his subject in a plan, common-sense manner, he tells to the school children a straightforward unvarnished tale about their teeth, and attempts to inculcate habits of cleanliness and hygiene which, if practiced, must stand the members of his young audience in good stead throughout life. As each child will, in time, occupy the position of the center of some household, the good teaching received will, in coming years, be capable of bearing a rich harvest. It is easy to impress upon the child's mind habits and thoughts which cling throughout life, and influence the acts of adult life. In England we care so little about school hygiene that it is a rare matter to find large schools to which any special dentist is attached, the poor children being given over to the ministration of the "doctor." The result of this arrangement is better imagined than described. In schools also where physiology is taught, which is seldom the rule, the little pupils are duly instructed concerning the development of the teeth, and are posted up with the latest theories about the enamel organ and dentine, but no mention is made of how important is the preservation of the teeth for the due carrying out of the vital process. It would be well if every large school, whether founded for the children of the well-to-do or for those less carefully nurtured, was placed in the hands of a competent dentist, who should not only treat his little patient's aching members, but who should be responsible for systematic instruction imparted to the pupils concerning the uses, the preservation, reparation and vital value of their teeth. Such teaching could be made very interesting and effective, and might be so given as to appeal directly to the children themselves, inducing them to emulate each other in a useful rivalry in cleanliness and general attention to their organs of mastication. We have insisted above upon the enormous value which must follow such instruction, and we have little doubt that before very long we shall see Mr. Moxon's very excellent example largely followed.

At present but few dentists have the chance of giving such lectures, but ere long the appointment of a dental surgeon to every workhouse, school, prison and reformatory must be an accomplished fact, and then a wider scope will be opened to the profession in this particular field of usefulness. One word of caution must, however, be uttered. It is above all things important that lectures should not be given for the sake of mere talking. Whatever is done in this direction will need to be carried through with tact and knowledge, singleness of purpose, and a sincere desire to benefit the audience. If any of these motives are lacking, by all means let popular lectures alone. It is true that such things may be most useful, but it is as irrefragable that they may be rendered baneful to the last degree.—*The British Journal of Dental Science.*

